# The evolution of human fatigue resistance

**DOI:** 10.1007/s00360-022-01439-4

**Published:** 2022-05-12

**Authors:** Frank E. Marino, Benjamin E. Sibson, Daniel E. Lieberman

**Affiliations:** 1 School of Allied Health, Exercise and Sport Science, Charles Sturt University, Bathurst, NSW 2795 Australia; 2 Department of Human Evolutionary Biology, Harvard University, 11 Divinity Ave, Cambridge, MA 02138 USA

**Keywords:** Adaptations, Endurance, *Homo*, *Pan*, Speed, Trade-offs

## Abstract

Humans differ from African great apes in numerous respects, but the chief initial difference setting hominins on their unique evolutionary trajectory was habitual bipedalism. The two most widely supported selective forces for this adaptation are increased efficiency of locomotion and improved ability to feed in upright contexts. By 4 million years ago, hominins had evolved the ability to walk long distances but extreme selection for endurance capabilities likely occurred later in the genus *Homo* to help them forage, power scavenge and persistence hunt in hot, arid conditions. In this review we explore the hypothesis that to be effective long-distance walkers and especially runners, there would also have been a strong selective benefit among *Homo* to resist fatigue. Our hypothesis is that since fatigue is an important factor that limits the ability to perform endurance-based activities, fatigue resistance was likely an important target for selection during human evolution for improved endurance capabilities. We review the trade-offs between strength, power, and stamina in apes and *Homo* and discuss three biological systems that we hypothesize humans evolved adaptations for fatigue resistance: neurological, metabolic and thermoregulatory. We conclude that the evolution of endurance at the cost of strength and power likely also involved the evolution of mechanisms to resist fatigue.

## Introduction

Although humans differ from other mammals in numerous respects, the fossil record supports Darwin’s 1871 speculation that a chief initial difference that set the human lineage on a separate evolutionary path from the apes was habitual bipedalism (Zollikofer et al. 2005; Richmond and Jungers 2008; Lovejoy et al. 1999). How and why habitual bipedal posture and locomotion evolved in hominins is the subject of intense debate. Among the many proposed selective forces thought to have favored the origin of habitual bipedalism, the two most widely supported by paleontological and experimental data are increased efficiency of locomotion (Rodman and McHenry 1980; Sockol et al. 2007; Pontzer et al. 2009) and improved ability to feed in upright contexts (Hunt 1994; Thorpe et al. 2007). Regardless of the initial driving forces selecting for bipedalism between 6 and 9 million years ago, hominins belonging to the genus *Australopithecus* between 3 and 4 million years ago were inhabiting relatively open habitats and were capable of striding walking gaits with relatively extended hips and knees (Ward 2002; Raichlen et al. 2008).

The ability to walk long distances efficiently likely helped australopiths forage for widely dispersed resources, such as underground storage organs (Laden and Wrangham 2005) and perhaps also avoid predation from predators during periods of peak midday heat (Lieberman 2015). Although daily travel distances for australopiths are unknown, they likely walked farther per day than extant African great apes. Chimps walk on average 2–4 km/day (Pontzer and Wrangham 2004) and gorillas typically travel less than 1 km/day (Goldsmith 1999). Short daily travel distances reduce energetic costs although transport costs in chimpanzees, measured as volume of muscle activated per meter, are approximately two times higher than in humans (Pontzer et al. 2009).

Further selection for endurance capabilities likely occurred during the evolution of the genus *Homo*. Given their similar body size, *H. erectus* hunter-gatherers probably walked similar distances as modern hunter-gatherers in tropical habitats, who average 9–15 km/day (Marlowe 2005). When walking, hunter-gatherers often carry substantial loads including children and food. By the time of *H. erectus*, hominins were also selected for the ability to run long distances to scavenge and persistence hunt in hot, arid conditions (Bramble and Lieberman 2004; Lieberman and Bramble 2007). Persistence hunts by modern hunter-gatherers typically involve a combination of walking and slow running over 15–30 km (Liebenberg 2006; Lieberman et al. 2020). Digging is another sustained physical activity that hunter-gatherer women often do for many hours a day (Marlowe 2010; Kraft et al. 2021).

Here we explore the hypothesis that for the genus *Homo* to be effective long-distance walkers and runners there would also have been a strong selective benefit to resist fatigue. More specifically, we hypothesize that there was selection for adaptations to resist fatigue, but also that some adaptations for endurance also help resist fatigue. Endurance can be defined as a general ability to sustain continuous whole-body movement, whereas fatigue resistance is related to those factors which can enhance endurance under specific situations (such as stress due to heat, metabolism, and cardiovascular demand). Although endurance and fatigue resistance appear to be synonymous, it is important to understand that endurance was selected to enhance our capacity to walk and run long distances, but could not have been possible without additional adaptations enhancing resistance to premature fatigue. For example, the ability to sweat and a variable metabolome would enhance fatigue resistance and in turn improve endurance. Fatigue resistance is difficult to test in non-human species but there is little disagreement that extant apes are more adapted for power rather than endurance (Lieberman 2021). In addition to lacking thermoregulatory adaptations to dump heat as effectively as humans, chimpanzee skeletal muscles are dominated by fast-twitch fibers (Thorpe et al. 1999; Payne et al. 2006; Myatt et al. 2011), and their hearts have small, relatively thick, trabeculated ventricles that compromise their ability to sustain high cardiac outputs for long durations (Shave et al. 2019). The primatologist Richard Wrangham has reported that chimpanzees never run more than ~ 100 m, and the longest distance he ever observed chimpanzees walk was 11 km, after which they rested for a full day (Wrangham, personal communication). Although an enhanced capacity for endurance is apparently a key derived human characteristic, there has been little research on how, or if, humans were selected to also resist fatigue during endurance activities. In addition, a greater understanding of the evolutionary bases of the human capacity to resist fatigue could provide new insights on the inherent limitations of human athletic performance.

**Fig. 1:**
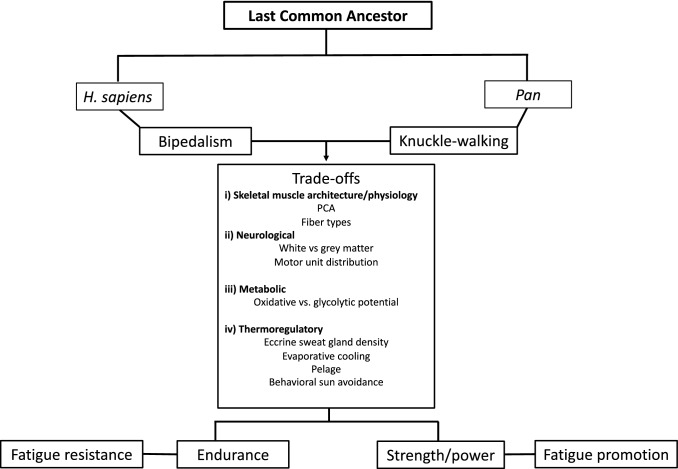
Schematic of the hypothesis that fatigue resistance was selected in parallel with adaptations for endurance in *Homo* compared with *Pan* which is more adapted for strength and power. Adaptions for fatigue resistance were likely precipitated by selection for habitual bipedalism that increased selection for trade-offs in skeletal muscle architecture/physiology, neurological, metabolic, and thermoregulatory functions. *H* is Homo, *PCA* is physiological cross sectional area

The purpose of this paper is, therefore, to consider fatigue processes in an evolutionary framework. We hypothesize that selection for endurance over power also led to selection for additional adaptations that enhanced fatigue resistance in humans compared with other primates. To make this argument, we first review the evidence for trade-offs between strength, power, and endurance in apes and hominins. We then discuss and compare hypothesized adaptations for fatigue resistance in three biological systems: neurological, metabolic and thermoregulatory. Figure 1 presents our theoretical proposition as a schematic.

## Evaluation of trade-offs between strength, power, and endurance

Before evaluating the evidence for selection for endurance over power and strength in hominins that potentially also favored selection for adaptations to enhance fatigue resistance, it is useful to first define and compare strength, power and endurance and the degree to which these capacities are trade-offs.

### Skeletal muscle architecture and physiology

Evolutionary theory predicts that organisms are subject to numerous trade-offs, because many resources are limited (e.g., one cannot spend a minute or a calorie twice) and because of physical constraints that make it impossible to increase or decrease two phenotypic traits simultaneously (Garland 2014). Three recognized and inter-related evolutionary constraints on physical performance are the apparent trade-off between skeletal muscle power with muscle endurance, the trade-off between power and strength, and the trade-off between strength and endurance. Muscle *endurance* is the capability to generate and maintain repeated or continuous non-peak forces for long durations (minutes to hours) without fatiguing (Wilkie 1960). From a physiological perspective, endurance is largely proportional to the physiological cross-sectional area (PCSA) occupied by slow-twitch, Type I fibers, as well as the density of mitochondria, capillaries, and myoglobin within muscle (Tsianos and Loeb 2017). Muscle *strength* is the maximum tensile force a muscle can generate and is largely proportional and relative to the PCSA occupied by fast-twitch, Type IIa and Iib fibers. Muscle *power* is the product of muscle force and muscle shortening velocity and is thus largely proportional to muscle PCSA and the length of muscle fibers, with longer fibers having more sarcomeres in series and thus able to shorten with higher velocity (Vogel 2001).

Physiological theory suggests that these differences and constraints in skeletal muscle properties make it impossible to enhance simultaneously muscle power, strength, and endurance. For example, only a limited amount of a muscle’s volume can be occupied by Type I and Type IIa and IIb muscle fibers (Garland and Carter 1994). Type I fibers generate less force, shorten with slower velocity, and have a higher density of mitochondria and myoglobin than Type IIa/b fibers, which generate higher levels of force, shorten more rapidly, and have fewer mitochondria and less myoglobin. As such, a muscle with more volume occupied by Type I fibers would be expected to have higher endurance at the cost of reduced power and strength, and vice versa. This predicted trade-off between endurance with power and strength has been experimentally studied in a range of species at different levels of biological organization, from single muscle fibers to whole organisms (Bennett et al. 1984; Garland 1988; Van Damme et al. 2002; Vanhooydonck et al. 2001; Wilson and James 2004; Wilson et al. 2002). A consistent finding is that at the single muscle fiber level there is a negative correlation between maximal power and strength with endurance as a function of fiber type composition. However, there is also a consistent lack of evidence to support a trade-off between speed, strength, and power with endurance at the organismic level (Wilson and James 2004). For example, an analysis of 600 world-class decathletes showed that individual performance in any pair of disciplines was positively correlated with the entire data set so the predicted trade-off between running speed (associated with strength, because the ability to run fast is determined largely by how forcefully an organism can strike its foot against the ground) and endurance was not detected (Van Damme et al. 2002). However, when restricted to individuals of comparable professional ranking, trade-offs between traits became evident. For example, among elite decathletes, performance in the 100 m sprint, shot put and long jump (activities requiring speed, strength, power) was negatively correlated with performance in the 1500 m run (an endurance event). In fact, a recent comparison between power vs. endurance trained athletes found that power production is traded for a higher cost of transport which was related to muscle fascicle length of the individual athlete’s muscles (Cooper et al. 2021). These and other data suggest that individual-level variation in overall athletic ability can mask trade-offs between endurance with strength, speed, and power, and high performance in one function may impede performance in other functions only when analyses are restricted to individuals of comparable athletic ability (Wilson et al. 2014; Lieberman et al. 2020). Notably, this trade-off is not restricted to humans, as a comparison of endurance vs. speed trade-off in frog muscle suggests that a physiological conflict between maximum power output and fatigue resistance exists at the level of vertebrate muscles (Wilson et al. 2002).

There are several mechanisms, whereby predicted trade-offs between endurance with strength and power may be bypassed at the whole organism level. One neuromuscular mechanism is to increase the number of muscle fibers that contract synchronously (Gorassini et al. 2002). Furthermore, while the total number of muscle fibers is generally constant, the size of individual muscle fibers can increase, both in terms of cross-sectional area and length (Abe et al. 2000; Hakkinen et al. 1998; Seynnes et al. 2007; Staron et al. 1991, 1994). Thicker muscle fibers contain more sarcomeres in parallel to increase force-generating capability, and longer muscle fibers have more sarcomeres in series to increase shortening velocity, hence power. Fiber type composition, muscle PCSA and fiber length are phenotypically plastic traits that depend on responses to varying doses of physical activities involving endurance *vs*. speed, strength, and power (Andersen and Aagaard 2010).

Physiological theory also predicts a trade-off between skeletal muscle strength and power. Although strength and power are not independent, because both are enhanced by a higher relative volume of Type IIa/b fibers within a muscle, strength derives more from a larger total muscle PCSA, whereas power derives more from longer muscle fiber lengths. The advantage of fiber length is related to the additional sarcomeres in series which results in less relative shortening per sarcomere (Jones et al. 2004). As a result, longer fibers have the capacity to shorten at a faster rate over a longer distance. It is also possible to increase both a muscle’s PCSA and fiber length with training. This has been shown in an avian model, whereby high acceleration training during the growth period increased the fascicle length in both extensor and flexor muscle by 12% and 14%, respectively, compared to sedentary controls (Salzano et al. 2018). According to the well-documented force–velocity relationship, muscle strength and power can never be co-optimized: muscles generate the most force when they are activated isometrically at their resting length or when they are briefly lengthened eccentrically (Kay et al. 2000), whereas muscles generate the most power when shortening depending on the stimulus frequency with higher frequencies producing higher power at a given velocity (De Haan 1998). An additional critical element for muscle power is efficiency which is determined by the relationship between relative and absolute shortening velocities. Between shortening velocities from zero to maximum, the efficiency rises to no more than 20–30% of maximum shortening velocity which corresponds to a load of 40–50% of maximum isometric force (Barclay 2019).

In sum, the theoretical predictions of trade-offs between endurance vs. strength/power and strength vs. power based on differences in fiber type composition, muscle PCSAs, and muscle fiber lengths are generally supported at the single muscle fiber level but not the whole organism level within species. Nevertheless, there is evidence that species which rely on extended periods of movement possess relatively large amounts of Type I fibers, whereas those that rely on bursts of speed possess relatively large amounts of Type II fibers (Rome et al. 1988; Oufiero et al. 2011; Vanhooydonck et al. 2001). Whether these tradeoffs reflect between species differences is more difficult to test, since there are additional factors, such as anatomical variations, motivation and a range of physiological adaptations to consider.

### Comparing endurance, strength, and power between *Pan* and *Homo*

Evaluating trade-offs between endurance, strength, and power in the context of human evolution also requires comparing these properties between humans and our closest relatives, chimpanzees (*Pan troglodytes*) and bonobos (*Pan paniscus*). Early studies attempting to measure the comparative strength of chimpanzees suggested chimps were 3–4 times stronger than human males when normalized for body mass (Bauman 1923, 1926). These results, however, should be treated with skepticism because of methodological problems, and lack of replication (Bozek et al. 2014; Edwards 1965) (Finch 1943). When scaled for body mass, bonobos can jump as high as humans (Scholz et al. 2006).

Comparisons of the fiber type composition of the triceps surae among orangutans and chimpanzees indicate a higher proportion of Type I fibers in orangutans, which likely corresponds to the slow and controlled arboreal quadrumanous locomotion they use in their forest habitats (Myatt et al. 2011). The higher proportion of Type IIa/b fibers found in chimpanzee triceps surae presumably reflects their greater need for speed, strength, and power-based physical activities, including sprinting and fighting. Despite much variation, the triceps surae averages ~ 50–70% Type I fibers (Edgerton et al. 1975).

While fiber type composition has a genetic component, it is also a phenotypically plastic trait that is influenced by stimuli, such as the types and doses of physical activities (Zierath and Hawley 2004). For example, the gastrocnemius muscle had equal proportions of Type I and Type IIa/b fibers in nonathletes, but ~ 73% Type IIa/b fibers in elite sprinters, and ~ 70% Type I fibers in long-distance runners (Costill et al. 1976a, b; Fink et al. 1977). Elite sprinters also tend to have larger lower limb muscles than long-distance runners and nonathletes, reflecting their need to generate high ground reaction forces (Handsfield et al. 2017).

A laboratory analysis conducted with fibers from 35 chimpanzee pelvis and leg muscles found that chimpanzee leg muscle fibers can produce ~ 30% more force and power than a typical human’s leg muscle fibers, with this difference explained by chimpanzee muscle having a higher proportion of Type IIa/b fibers; unlike humans, chimpanzee muscle was composed of ∼67% Type II a/b fibers (O’Neill et al. 2017). Chimpanzee muscle fibers were also longer, enabling them to shorten with higher intrinsic velocity and thus generate more power. These findings, combined with the findings of the several “pulling strength” studies discussed earlier comparing chimpanzees and humans, suggest that chimpanzees are about 1.3–1.5 × stronger than humans (Lieberman 2021).

In addition to interspecific differences in fiber type composition, chimpanzees have almost twice as much muscle mass in the forelimbs compared to humans, whereas humans have significantly more muscle mass in the hind limbs (~ 250 g kg^−1^ body mass in humans vs. 170 g kg^−1^ body mass in chimps) (Thorpe et al. 1999; Payne et al. 2006). Although human hind limb muscles have larger mass and PCSA, they also have shorter fascicle lengths. Specifically, humans have more sarcomeres in parallel, which combined with shorter fascicle length allows for the generation of large moments around a joint at the cost of limited range of motion and speed, hence reduced power. See Table 1 (Thorpe et al. 1999; Zihlman 1992; Payne et al. 2006; Tirrell et al. 2012; Yamaguchi et al. 1990; Simoneau and Bouchard 1989; Bozek et al. 2014; O’Neill et al. 2017) for interspecies comparisons of anatomical and physiological traits related to strength, endurance, and power.

**Table 1:** Differences in skeletal muscle architecture and physiology accounting for the disparity in strength, power and endurance between humans and chimpanzees

Species	Forelimb muscle mass (% body mass)	Hindlimb muscle mass (% body mass)	Muscle fascicle length	Muscle PCSA (cm^2^)	Hindlimb type I muscle fiber %	Hindlimb type IIa muscle fiber %	Hindlimb type IIb muscle fiber %
*Homo sapiens*	9%^b^^,^^f^	250 g kg^−1^; 38%^c^	Short	Large	48.5–69.0^g,h,i^	38.0^d^	13.5^d^
*Pan troglodytes*	16%^b^^,^^f^	170 g kg^−1^; 24%^c^	Long	Small	34.4–43.8^e,i^	33.7 (7.2)^e^ 18.3^d^	31.8 (1.4)^a^ 34.7^d^

Percentage represents distribution of total body mass located in that region (forelimbs or hindlimbs). PCSA is the relative physiological cross-sectional area when corrected for total body mass. Muscle fiber type % values are from vastus lateralis muscle fibers

^a^Thorpe et al. (2007)

^b^Thorpe et al. (1999)

^c^Payne et al. (2006)

^d^Brozek et al. (2014)

^e^O’Neill et al. (2017)

^f^Zihlman (1992)

^g^Tirrell et al. (2012)

^h^Yamaguchi et al. (1990)

^i^Simoneau and Bouchard et al. (1989)

Humans are habitual long-distance bipedal walkers and runners, while chimpanzees are knuckle walkers who engage in short bouts of vertical climbing and otherwise engage in suspensory locomotion. Given that the lumbar region of humans is long, curved, and mobile compared to the entrapped, short, and straight lumbar region of chimpanzees (Pilbeam 2004; Thompson and Almécija 2017), it is reasonable to expect there might also be differences in trunk muscle architecture and physiology between species. When comparing fiber type composition of the lumbar muscles of gibbons, orangutans, bonobos, and chimpanzees, each of these hominoids had a generally “homogeneous” distribution of fiber types (similar amounts of Type I and IIa/b fibers spread evenly throughout the cross section of a muscle) among both deep and superficial muscles, with orangutans and gibbons having overall higher proportions of Type I fibers (Neufuss et al. 2014). Whether the fiber type distribution of human lumbar muscles also displays this “homogenous” pattern, and whether the overall proportion of fiber types in human lumbar muscles is more skewed towards Type I or Type IIa/b fibers, has yet to be comprehensively tested. Hesse et al. (Hesse et al. 2013) reported that the lumbar muscles of 2 male human cadavers aged 64 and 95 years had a homogeneous distribution of fiber types with no deep-superficial functional distinction, but this result is limited by the small sample size. Neufuss et al. (2014) suggest that the similar fiber type composition of the lumbar muscles of humans, chimpanzees, and bonobos reflects similar evolved functions of the lumbar muscles among these species. However, other studies that measured the fiber type composition of human lumbar muscles report a wide range and variability of values, with some muscles (e.g., multifidus and longissimus lumborum) being composed of more than 90% Type I fibers in some samples (Mannion et al. 1997; Ng et al. 1998; Tirrell et al. 2012). Given the endurance-based locomotion of humans and the power-based locomotion of chimpanzees and bonobos, we hypothesize that, consistent with the muscles of the lower limb, human lumbar muscles may have evolved to have a higher Type I fiber composition than those of chimpanzees and bonobos. Type I fiber dominance seems desirable, since adaptability for fatigue resistance for constant bipedal locomotion rather than climbing would be an advantage for *Homo*. However, more data are needed on the fiber type compositions of human, chimpanzee, and bonobo lumbar paravertebral muscles to test this hypothesis.

In sum, interspecific differences in muscle size, fiber type composition, and fiber length indicate that chimpanzees are adapted for climbing and sprinting behaviors that require power and strength, whereas humans are adapted primarily for long-duration bipedal walking and running behaviors that require endurance.

## Hypothetical adaptations for fatigue resistance in *Homo* and non-human primates

The hypothesis that selection for endurance included selection for fatigue resistance predicts that adaptations for fatigue resistance differ between humans and non-human primates, especially the African great apes. Unfortunately, the comparisons necessary to test this prediction are limited by lack of data from non-human primates. We are thus constrained to focus on the available evidence for mechanisms of fatigue resistance in humans in three systems: nervous, metabolic and thermoregulatory. We emphasize that many of these are hypothesized adaptations that need to be tested with additional comparative data from non-human primates.

### Potential neurological adaptations for fatigue resistance in *Homo*

One potential mechanism that may explain the higher strength and power of apes compared with the higher endurance of humans, potentially relevant for fatigue resistance, could be differences in neuronal arrangement, from the CNS to the skeletal muscles. Early studies (MacLarnon 1995, 1996) comparing the relative quantity of white and gray matter in the CNS found that compared with humans, chimpanzees had a higher amount of white matter relative to gray matter in their spinal cords when corrected for spinal cord cross-sectional area and normalized to body mass. Since neuronal white matter increases nerve conduction velocity, we predict that chimpanzees have comparatively higher numbers of large, fast-conducting motor units that innervate Type IIa/b muscle fibers. Conversely, we also predict that humans would have a comparatively wider distribution of motor units which allows for finer motor control at the cost of less muscle force and slower shortening velocities (and, therefore, less power but more endurance and fatigue resistance) (Sale 1987).

In this context it is useful to note that the distribution of fiber types is neither random nor uniform but varies from deep to surface areas of the muscle. For example, in human *vastus lateralis* there is a predominance of Type II fibers at the surface and Type I fibers in deeper regions (Lexell et al. 1983), which is the opposite for the *anterior tibialis* (Henriksson-Larsén et al. 1983). Although there is scant comparative data on lower limb muscles for non-human primates, it is possible that a similar distribution to the *vastus lateralis* in humans exists in other non-human primates based on the Cynomolgus (crab-eating macaque) monkey leg and thigh extensor compartments (Acosta and Roy 1987); Type I fibers are concentrated in deep regions vs. Type II fibers concentrated in superficial regions. Since fiber type distribution does not seem to be random (even when homogeneously distributed), these authors postulated that this distribution reflects the functional demands, whereby different parts of muscle will be used during different phases of movement (such as postural activities vs. speed-based or explosive movements). It is not entirely clear why this distribution is favored either within humans and chimpanzees or between species. However, since the only existing evidence suggests that chimpanzees have a higher amount of white matter relative to gray matter in the spinal cord, it is a reasonable assumption that the proportion of fiber types favoring strength and power will be consistent with their fiber distribution within a particular muscle.

Another aspect of the neuromuscular system that favors endurance and possibly also fatigue resistance in humans, and was potentially under selection is the size of small motor units with low action potential-thresholds that innervate Type I fibers (Stålberg and Fawcett 1982). The total number of motor units is known to decrease with age (Piasecki et al. 2016), which can result in complete skeletal muscle activation and reduced efferent drive (Unhjem et al. 2016). Given that muscles with high proportions of Type I fibers generally function to maintain posture and stabilize joints, and these functions stay vital with aging, it may be the case that the increase in size of the slow motor unit pool functions to preserve mobility and physical activity into old age. This makes sense from an evolutionary anthropological perspective, as older adults in hunter-gatherer and subsistence farming societies stay active throughout their lifespans, continuing to walk, dig, and carry into their 60 s and sometimes into their 70 s and 80 s (Lieberman et al. 2020). Currently there are no data on age-dependent changes in motor unit size and number in chimpanzees, although in a recent study on aging and frailty in wild chimpanzees, older males and females were found to spend less time in trees compared with their younger counterparts (42 and 26 min less, respectively, per 12 h day). Although older chimps have reduced lean muscle mass, sarcopenia was not a significant factor in predicting the trend for time spent in trees/day (Thompson et al. 2020). Whether this reduced lean muscle mass favors a particular fiber type remains to be tested. These data are also potentially confounded by the possibility that ageing is associated with reduced caloric intake, and therefore, reduced arboreal time might reflect this rather than specific alterations in muscle fiber type and/or distribution.

### Potential metabolic adaptions for fatigue resistance in *Homo*

Selection also appears to have acted on several aspects of human metabolism that enhance endurance at the expense of strength and power that may also be relevant for considering fatigue resistance. When comparing the *vastus lateralis* of humans and chimpanzees, human skeletal muscle has a derived metabolic profile indicating that metabolome evolution in skeletal muscle was accelerated in the hominin lineage (Bozek et al. 2014). Given the reduced strength of the *vastus lateralis* in humans compared to chimpanzees, it was hypothesized that the accelerated metabolite evolution and reduced muscle strength in humans could be explained by interspecific differences in fiber type composition. Different fiber types have different metabolic profiles: compared to Type IIa/b fibers, Type I fibers have higher oxidative potential, reduced glycolytic potential, lower concentrations of phosphocreatine and glycogen, and higher concentrations of triglycerides (Zierath and Hawley 2004). To partially evaluate their hypothesis, Bozek et al. (2014) measured and compared fiber type composition of the *vastus lateralis* of nine chimpanzees and found the proportion of Type I fibers to be similar to a sample of human *v. lateralis* (48.5% in humans vs. 43.8% in chimps) (Simoneau and Bouchard 1989). The authors interpreted this as evidence that interspecific differences in fiber type composition likely do not alone explain the metabolic divergence of human skeletal muscle. Rather, there appear to be accelerated changes in the metabolite concentrations in the prefrontal cortical region of the brain and skeletal muscle of humans. It is entirely possible that the allocation of the metabolome in the brain tissue represents the selection of muscle composition based on use. However, in their sample humans had a much higher proportion of Type IIa fibers (38 vs. 18.3%) and a much lower proportion of Type IIb fibers than chimpanzees (13.5 vs. 34.7%). Type IIa fibers fall between Type I and IIb fibers in terms of contractile characteristics and oxidative and glycolytic potential. Notably, O’Neill et al. (2017) in comparing the strength of chimpanzees and humans found that the higher strength of chimpanzees can be accounted for by the higher proportion of myosin heavy chain (MHC) isoform. These findings are in line with the understanding that MHC is key for the development of skeletal muscle force and contraction velocity. The combined findings from Bozek et al. (2014) and O’Neill et al. (2017) on fiber type composition in humans and chimpanzees, suggest real and meaningful evolved differences in fiber type composition and metabolic profiles of the skeletal muscles of humans compared to chimps. We encourage further testing of the hypothesis that these evolved differences in fiber type composition are associated with the accelerated metabolite divergence of human skeletal muscle.

### Potential thermoregulatory adaptations for fatigue resistance in *Homo*

The thermoregulatory capacity of humans has been well documented in a range of contexts and environments (Blake and Larrabee 1903; Adolph 1947; Ladell 1957; Marino 2018). Thermoregulatory capabilities have also been compared between humans and many other taxa including cheetah (Taylor and Rowntree 1973), African hunting dog (Taylor et al. 1971), goats (Caputa et al. 1986), horses (Geor et al. 1995) and rodents (Fuller et al. 1998). There is strong support for the hypothesis that members of the genus *Homo* evolved numerous adaptations to thermoregulate effectively and efficiently in hot, arid climates to withstand the environmental challenge of foraging in the heat of the day when carnivores are less active and to hunt when human thermoregulatory capabilities give running hunters an advantage during persistence hunting (Carrier et al. 1984; Bramble and Lieberman 2004; Liebenberg 2006; Lieberman 2015). Due in part to increased eccrine sweat gland density over the whole body combined with the lack of fur, humans dump heat more effectively than other mammals and primates over long periods, during vigorous physical activity.

Human abilities to dissipate heat by sweating, combined with a bipedal gait are also relevant to resisting hyperthermia-induced fatigue. A pursued quadruped can either trot or gallop to escape. The advantage of trotting is that it avoids hyperthermia and fatigue, but the gait is slow and increases the risk of being captured by a faster predator. Galloping is faster and can put distance between prey and predator, sometimes affording a rest period before a subsequent chase. This strategy is more thermogenic and sharply increases the risk of hyperthermia, because galloping quadrupeds cannot pant, which is their primary means of evaporative heat loss (Bramble and Jenkins 1993). In contrast, bipedal humans can decouple respiration and gait when running (Bramble and Carrier 1983; Callison et al. 2019), and also have enormous capacity for evaporative cooling through profuse sweating. Humans can sustain sweat rates up between 2 and 4 l/h (Pugh et al. 1967; Armstrong et al. 1986), which is about 2–3.4 times greater than eland, which have a better capacity to sweat than most other quadrupeds (Taylor and Lyman 1972). Since sustaining long periods of vigorous physical activity in the heat relies on the capacity to dump heat, humans thus have a thermoregulatory advantage over other quadrupeds, enabling humans to engage in persistence hunting not just in hot arid desert and savannah habitats, but also in temperate habitats during the summer (for review, see Lieberman et al. (2020)).

Among humans, larger individuals have proportionately higher rates of heat storage when ambient temperature rises above 25 °C and %*rh* exceeds 60%, which can result in core temperatures (*T*_c_) more than 39.5 °C (Marino et al. 2004). Although this level of hyperthermia is widely thought to lead to premature fatigue, field data from mass participation of runners in a 21 km road race indicates that 39.5 °C might not be a limiting value (Byrne et al. 2006; Lee et al. 2010), suggesting there are as yet untested mechanisms of fatigue resistance. These authors reported that a critical *T*_c_ in the range of 39.5–40.4 °C is not associated with fatigue or exhaustion, since two-thirds of the *T*_c_ responses were > 39.5 °C, and none of those runners succumbed to exhaustion or limiting fatigue. In fact, mean running speed of the 12 hyperthermic runners was not different before or after reaching a *T*_c_ of 39.8 °C. Notably, the fastest runner initiated a marked increase in speed over the final kilometer, when *T*_c_ was 40.6 °C, reaching 40.9 °C at race completion. The physiological mechanisms for this apparent capacity to withstand high *T*_c_, resist fatigue, and to increase pace are not well understood. Nevertheless, these data do underscore the human capacity to resist fatigue even when hyperthermic and provide an enticing avenue for future research into mechanisms of fatigue resistance.

In contrast to the evidence on human abilities to resist fatigue when hyperthermic, data on this ability in non-human primates are scarce. While eccrine sweat glands are present on the palmar surface of all mammals, presumably as an adaptation to increase friction during escape, and Old World Monkeys have eccrine glands throughout their bodies, there is a tenfold increase in eccrine gland density that is derived in humans compared to chimpanzees and macaques (Kamberov et al. 2018). In addition, humans have enlarged eccrine glands with higher per-gland sweat production (Best and Kamilar 2018). Experimental evidence does indicate that chimpanzees sweat, primarily around the axillae and groin, but not as profusely or effectively as humans (Whitford 1976). In addition, it has been claimed that chimpanzees have difficulty maintaining a constant body temperature and can succumb to heat stroke at ambient temperatures of ~ 40 °C (Whitford 1976), which is not normally the case with humans (see above). It is also noteworthy that non-human primates can increase their respiratory frequency to lose heat by panting (Hiley 1976).

An additional thermoregulatory disadvantage for chimpanzees and other non-human primates relevant to fatigue is pelage. In humans there has been a transition towards microscopic (vellus) hair on most of the body (except the scalp, groin, and armpits), which increases airflow at the skin surface, permitting effective evaporation, hence cooling. Therefore, it is not surprising that in both the wild and in captivity, chimpanzees and other mammals effectively thermoregulate behaviourally using sun-avoidance strategies, such as seeking shade, moving to the ground, and reducing their physical activity level (Kosheleff and Anderson 2009; Duncan and Pillay 2013). Since non-human primates are mostly terrestrial quadrupeds, their habitual stance exposes a larger surface area to solar radiation and potentially increased heat storage and suggested that bipedalism is partly an adaptation to reduce this exposure (Wheeler 1984, 1994).

## Conclusions

Our main hypothesis is that selection for endurance over strength and power in hominins, which appears to have occurred by the time of *Homo erectus*, resulted in concomitant selection for an improved capacity to resist fatigue in the neurological, metabolic, and thermoregulatory systems. The primary shift which set the stage for adaptations to improve endurance and fatigue resistance was the evolution of habitual bipedalism, which favored extended periods of upright feeding, long-distance walking, and eventually running, especially in more open, drier habitats than typically occupied by the African great apes with which hominins share a last common ancestor.

Although physiological trade-offs between endurance and strength and power are well established, there is abundant evidence that strength and power training can also improve endurance capacity and fatigue resistance. The reasons for this effect are not particularly obvious, although one enticing suggestion is that our survival in energy-scarce environments constrained our evolution for endurance, and in so doing endowed us with skeletal muscle plasticity so that improvements in endurance, and hence fatigue resistance could be gained through an additional mechanism (Best 2020). Thus, the endurance-strength/power trade-off is skewed toward endurance in humans, which also involved the evolution of mechanisms to resist fatigue. However, there are times of apparent voluntary override of perceived fatigue, although it is uncertain what specific biological limitations are being bypassed or overcome during the fatigue process to make this possible. Therefore, we encourage research that studies specific interventions and pathologies related to fatigue as well as fatigue resistance with the goal of progressively understanding the contributions that different parts of the process play in the overall result.

Finally, a less studied or considered area of fatigue is the contribution of mental fatigue. We are only just beginning to understand the possible additive effect of mental fatigue in determining human function and performance under various conditions (Marcora et al. 2009; Marcora and Staiano 2010). Mental fatigue can and does alter function and performance based on whether the activity involves endurance or power, and mental and physical fatigue are arguably separate, yet connected, phenomena. Studying mental fatigue provides an enticing avenue to further understand the fatigue process in health, evolution, and disease (Staiano et al. 2018).
